# Trend, disparities, and projection analysis of public data on foot fractures in Sweden: a retrospective analysis of 179 129 fractures

**DOI:** 10.1186/s12891-024-07711-8

**Published:** 2024-07-27

**Authors:** Michael Axenhus, Martin Magnéli

**Affiliations:** 1 Department of Orthopaedic Surgery, Danderyd Hospital, Stockholm, Sweden; 2 Department of Clinical Sciences at Danderyd Hospital, Karolinska Institutet, Stockholm, Sweden

**Keywords:** Age and sex disparities, Epidemiology, Preventive measures, Foot fractures, Trends

## Abstract

**Introduction:**

Orthopedic injuries to the foot constitute a significant portion of lower extremity injuries, necessitating an understanding of trends for effective preventive strategies and resource allocation. Demographic shifts, improved traffic safety, and lifelong physical activity may alter incidence rates, trauma mechanisms, and fracture distribution. This study explores the prevalence of foot fractures in Sweden using publicly available data.

**Methods:**

Utilizing data from the Swedish National Board of Health and Welfare (SNBHW) spanning 2008–2022, retrospective study focuses on foot fractures in Sweden. Analysis includes calculating annual incidence rates per 100,000 person-years, assessing temporal trends, and exploring seasonal variations. Poisson regression analysis was used for projections into 2035.

**Results:**

Between 2008–2022, the average annual foot fracture incidence was 11,942, with notable fluctuations influenced by the COVID-19 pandemic. Age and sex disparities impact rates, and seasonal variance highlights increased incidence in summer. By 2035, foot fractures will decreasae amongst several demographic groups.

**Conclusion:**

This study provides insights into temporal trends, sex differences, and seasonal variations foot fracture patterns in Sweden. The identified trends suggest the utilization of targeted preventive strategies, efficient resource allocation, and informed healthcare planning. Despite limitations, this research offers valuable insights into foot fractures within the Swedish population, utilizing publicly aggregated data.

## Introduction

Orthopedic injuries involving the foot are widespread in clinical settings and account for almost 40% of all lower extremity injuries [1, 2]. These injuries can have a profound impact on individuals’ well-being and autonomy. Understanding the trends and nuances associated with foot fractures is imperative for the development of effective preventive strategies, optimal allocation of healthcare resources, and enhancement of overall care quality.

Foot fractures, almost always resulting from traumatic events, are influenced by a myriad of factors such as diminished bone density, muscle weakness, balance issues, and age-related physiological changes [3, 4]. Beyond inflicting physical discomfort and functional limitations, these fractures heighten the risks of hospitalization, disability, and mortality [5–7]. Despite the availability of successful treatments like surgery or casts, concerted efforts should be channeled towards curbing the incidence of foot fractures due to the substantial economic burden on healthcare systems arising from associated costs like hospital admissions, surgical procedures, rehabilitation, and long-term care [8–10].

Although ankle fractures are relatively well studied, foot fracture excluding the ankle are less explored in terms of incidence in age groups and the literature lacks a complete understanding of the epidemiology of foot fractures in various age groups. Furthermore, the identification of patient populations at risk is important in order to facilitate targeted preventative measures, women and elderly have been suggested as particularly vulnerable groups in terms of foot fracture incidence [11, 12]. Trend analysis studies also show that the incidence of foot fractures are expected to increase significantly and pose a serious burden on healthcare systems [13, 14]. Future trend prediction can therefore be useful for help stakeholders and healthcare systems in order to properly allocate resources to meet healthcare demand.

Sweden, like many other nations, grapples with the ramifications of an aging population. It is therefore crucial to monitor and analyze trends in foot fractures within the population to pinpoint high-risk groups, tailor preventive interventions, and efficiently allocate healthcare resources. Open datasets could be a useful approach to monitor foot fracture incidence due to their availability.

The aim of this study is to examine trends in the incidence of foot fractures in Sweden, with a specific focus on sex, age, and temporal disparities, utilizing publicly available data. By describing trends in foot fractures, valuable insights into the epidemiology of foot fractures can be derived which might aid in the identification of areas for intervention, prevention, or groups at risk.

## Methods

### Study design and data source

In this retrospective population-based study, data were sourced from the Swedish National Board of Health and Welfare (SNBHW) [15]. The SNBHW maintains comprehensive records of hospital admissions, outpatient specialist visits, and diagnoses for all individuals receiving treatment in Swedish hospitals. The SNBHW diagnosis register encompasses data on all diagnoses in Sweden, including cause of death recorded in the national patient register (NPR). These national registers ensure thorough coverage of healthcare utilization and mortality data, facilitating the analysis of foot fractures within the population. The NPR provides publicly aggregated data on a population basis, reporting ICD-10 codes per unique personal identification number only once per year and diagnosis group, minimizing the risk of duplicate reporting. Population census data was obtained from Statistics Sweden [16].

### Study population

This study focused on individuals residing in Sweden who experienced foot fractures during the time periods of 2008–2022 and 2008–2019. Data from two distinct periods were included, specifically from January 1st, 2008, to December 31st, 2021, and January 1st, 2008, to December 31st, 2019. The choice of two different time periods aimed to account for the impact of the COVID-19 pandemic on diagnostics. This selected timeframe allowed for a comprehensive analysis of temporal trends in foot fractures over a sufficiently extended period for future trend projection.

Identification of foot fractures was based on the International Classification of Diseases, Tenth Revision (ICD-10) codes [17]. All fractures of the foot, including fractures of the tarsus and metatarsals (ICD-10 codes S92) were included, and relevant cases were extracted from the NPR diagnoses register, a total of 179,129 cases during the study period.

We stratified the data in patients under and over 65 years of age. We chose the age of 65 as a cutoff point due to the higher incidence of fractures in this population owing to lower bone density [18, 19]. Patients under 18 years old were excluded. Age-stratified data was segmented into months to detect seasonal variances in the incidence of foot fractures among different age groups. Seasonal variations were reported in four seasons (Winter: December–February, Spring: March–May, Summer: June–August, Fall: September–November). A P-value of < 0.05 was considered statistically significant. Additionally, stratified analyses by sex were conducted to explore potential differences in foot fracture rates.

### Data analysis

Overall annual incidence was calculated per 100,000 person-years and stratified according to sex and age groups. Incidence rates were computed by dividing the number of foot fractures by age-specific population rates, using weights derived from the population distribution of standard population estimates. These estimates were obtained from Statistics Sweden [16]. The annual incidence rates of foot fractures were calculated per 100,000 person-years. Poisson regression analysis was conducted to project future trends. The model included year, age group and sex as predictors. Using historical data (2008–2021), we fitted the model to estimate predictor coefficients. Predictions for 2025, 2030, and 2035 were made by applying these coefficients to future values of the predictors. Separate analyses excluded data from 2020–2021 to account for COVID-19's impact, ensuring projections were not skewed. A *p*-value of < 0.05 was considered significant. All calculations were carried out using SPSS (Version 25.1).

### Ethical considerations

The reporting of the study complies with the Strengthening the Reporting of Observational Studies in Epidemiology (STROBE) statement [20]. The data used in this study were publicly available, anonymized, de-identified, and therefore not subject to ethical review.

## Results

### Fracture incidence

**Fig. 1:**
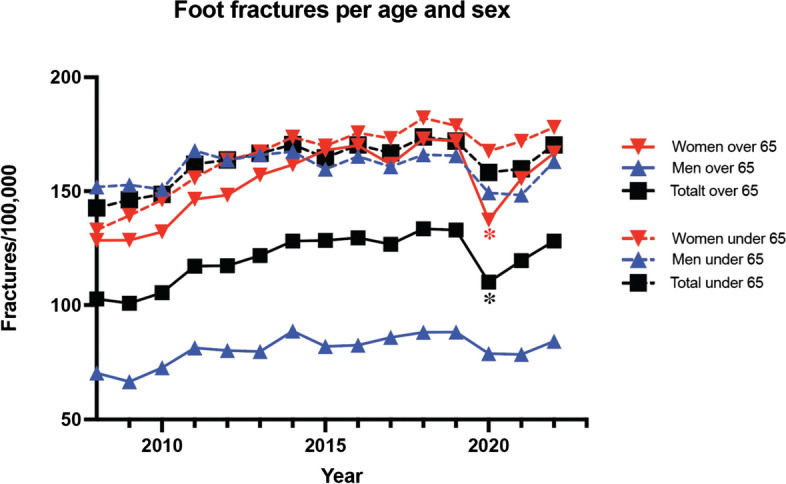
Age and sex distribution of foot fracture incidence rates in Sweden 2015–2021. Red asterisk indicates *p* < 0.05 for women compared to previous years. Black asterisk indicates *p* < 0.05 for total value compared to previous years

Between 2008 and 2022 there were an average of 11 942 patients registered with a foot fracture per year in Sweden. There was a significant drop in incidence during 2020 with a rebound of fractures during 2021–2022 (Table 1). The reduction in fracture incidence during 2020 was significant for women over 65 (*p* < 0.001) and men and women under the age of 65 years (*p* = 0.002) (Fig. 1).

**Table 1:** Fracture number, fracture incidence rates and expected fracture incidence amongst men and women during 2008–2022

	**Patients (n)**			**Incidence rate/100,000**		
**Year**	**Men**	**Women**	**Both sexes**	**Men**	**Women**	**Both sexes**
2008	4 897	4 901	9 798	135,4	131,9	133,6
2009	4 947	5 131	10 078	135,1	136,6	135,8
2010	4 978	5 411	10 389	134,4	142,7	138,6
2011	5 578	5 866	11 444	149,1	153,4	151,3
2012	5 486	6 150	11 636	145,3	159,6	152,5
2013	5 584	6 390	11 974	146,5	164,6	155,7
2014	5 755	6 667	12 422	149,4	170,5	160,0
2015	5 512	6 669	12 181	141,8	169,4	155,6
2016	5 764	6 920	12 684	146,2	174,1	160,2
2017	5 716	6 821	12 537	143,3	170,1	156,7
2018	5 956	7 260	13 216	147,7	179,6	163,7
2019	6 002	7 205	13 207	147,3	176,9	162,1
2020	5 430	6 522	11 952	132,6	159,3	145,9
2021	5 432	6 905	12 337	131,5	167,4	149,5
2022	6 004	7 270	13 274	144,0	174,9	159,4

### Age and sex distribution

**Fig. 2:**
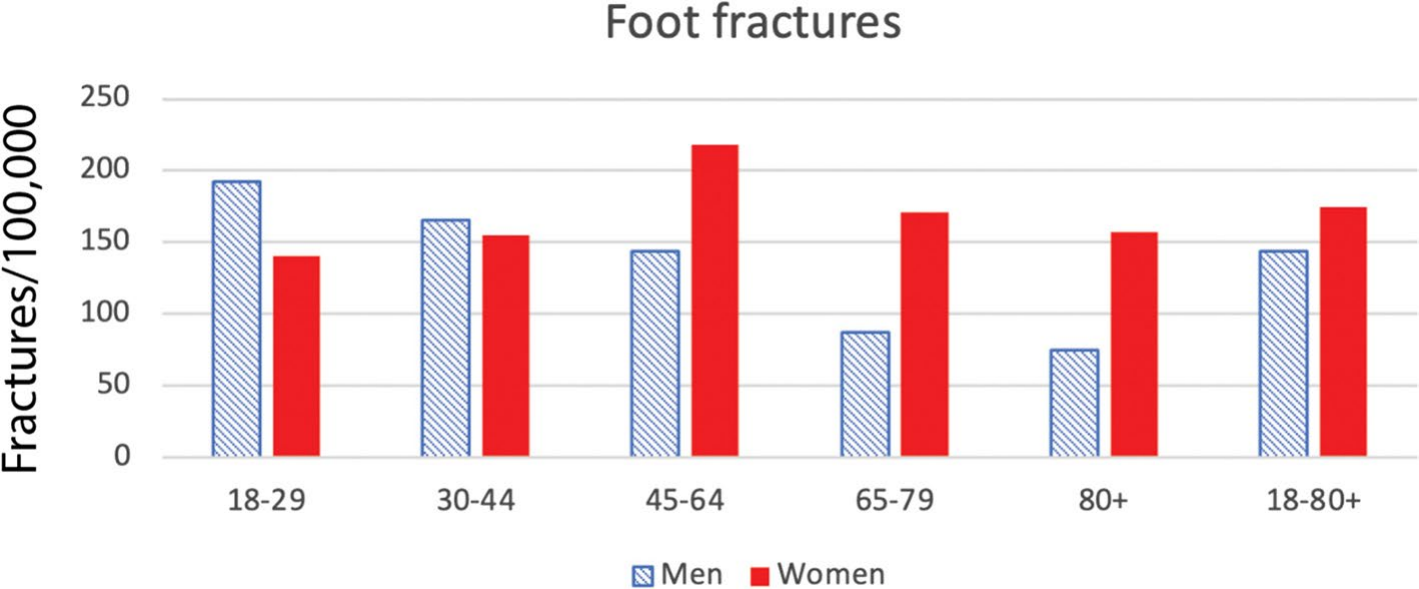
Age distribution of foot fractures in Sweden during 2022

Age- and sex-specific incidence rates per 100,000 person-years of foot showed that women experienced more foot fractures than men, particularly in the elderly categories (Fig. 2). In women, the incidence of fractures reached a peak of 218/100,000 per inhabitants in the 45–64 age group. In men, incidence was the highest in the 18–29 age group at 140/100,000 per inhabitants.

### Seasonal variance in fracture incidence

**Fig. 3:**
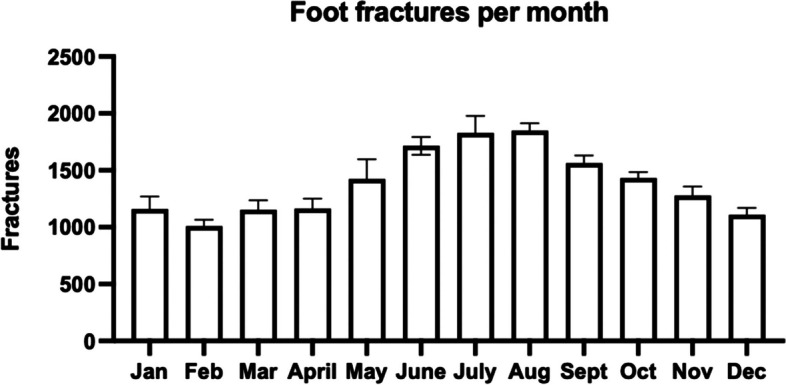
Average monthly mean of foot fractures during 2008–2022

Cumulative data demonstrated increased incidence in the summer months (May-Aug) (Fig. 3).

**Fig. 4:**
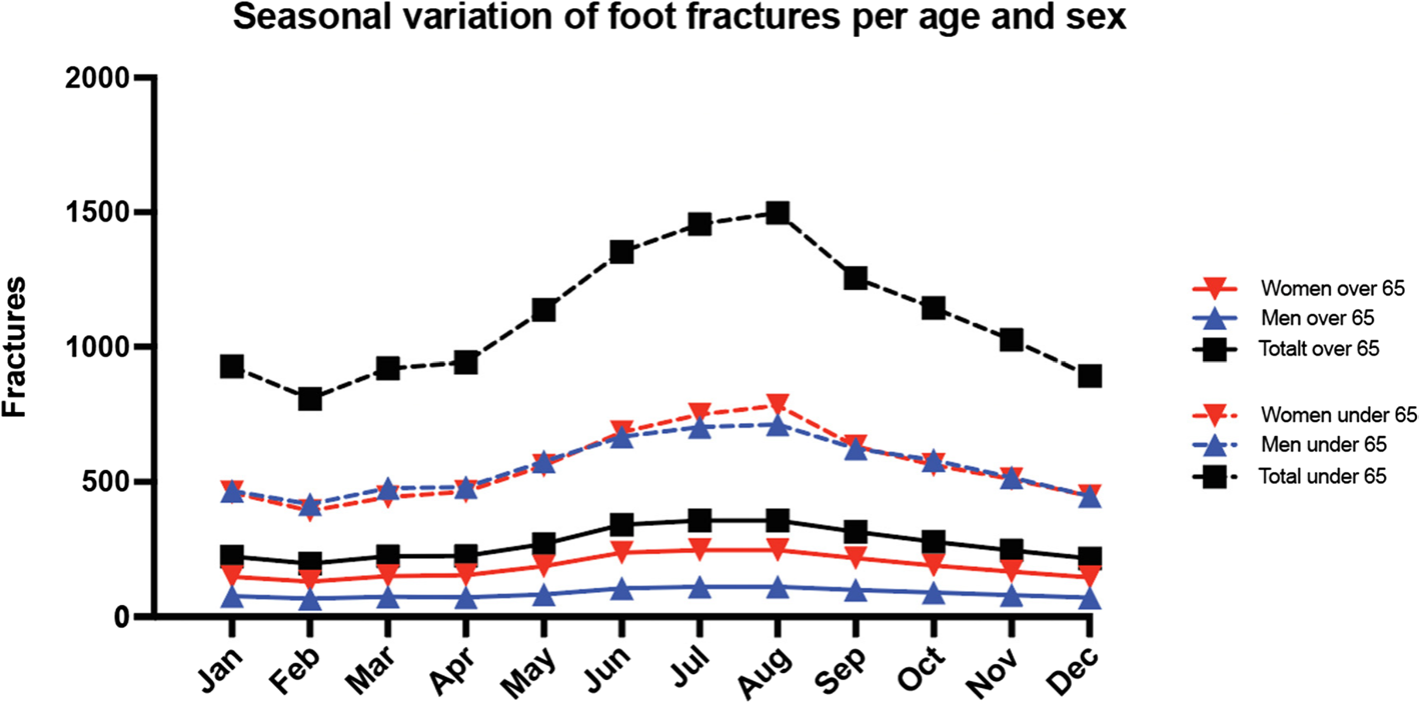
Seasonal variance in foot fractures per age and sex. Average mean during 2015–2022

Fracture incidence rate tended to decrease during autumn and late spring (Fig. 4). Across the seven-year period, 40% of fractures occurred in summer compared with 20%, 22% and 18% in spring, autumn and winter, respectively. Men and women under the age of 65 experienced more foot fractures compared to older groups. All groups registered more foot fractures during the summer months.

### Fracture projection analysis

**Fig. 5:**
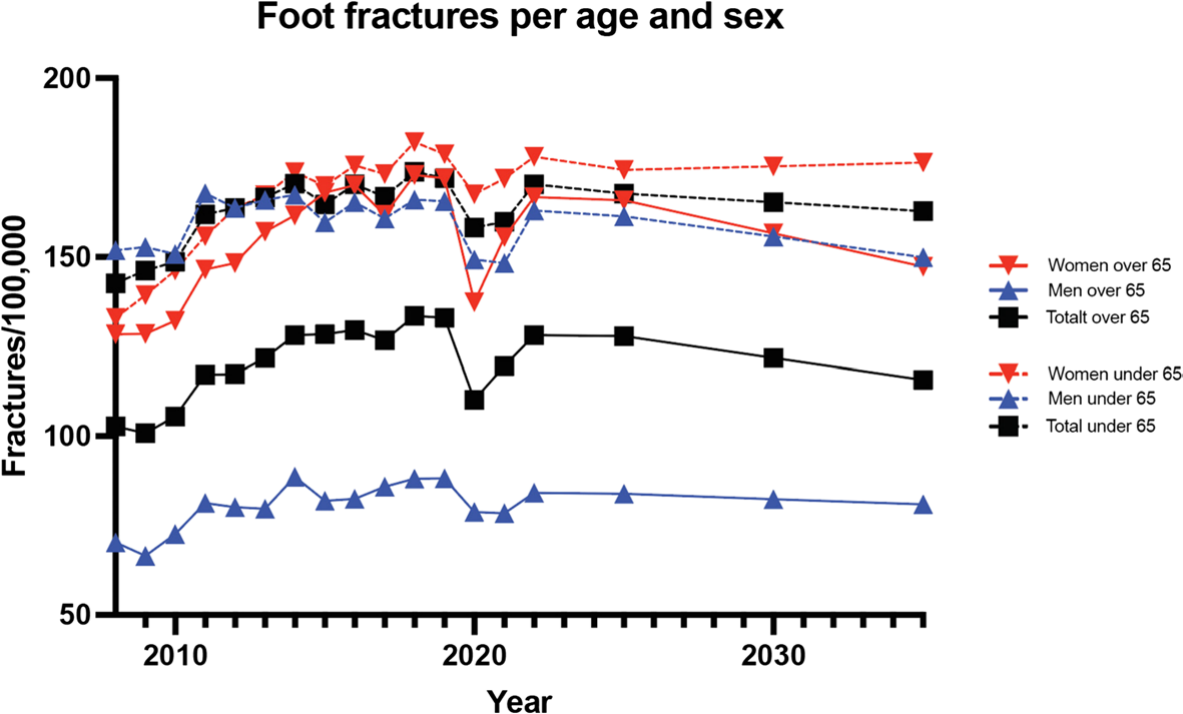
Projection of the incidence of foot fractures in men and women

We performed temporal trend prediction for 2025, 2030 and 2035. By 2035, the incidence of foot fractures will decrease in both the elderly and the younger category (Fig. 5). Women under the age of 65 will see an increase in foot fracture incidence (Table 2).

**Table 2:** Projected change in foot fracture incidence amongst men and women until 2035. 2022 value is displayed as fractures per 100,000. 2025–2035 shows expected change in incidence compared to 2022

	Men over 65	Men under 65	Women over 65	Women under 65	Total over 65	Total under 65
Year						
2022	84,2	163	166,7	178	128,1	170,3
2025	99%	97%	99%	99%	99%	98%
2030	98%	94%	95%	102%	96%	98%
2035	97%	91%	89%	105%	93%	96%

**Fig. 6:**
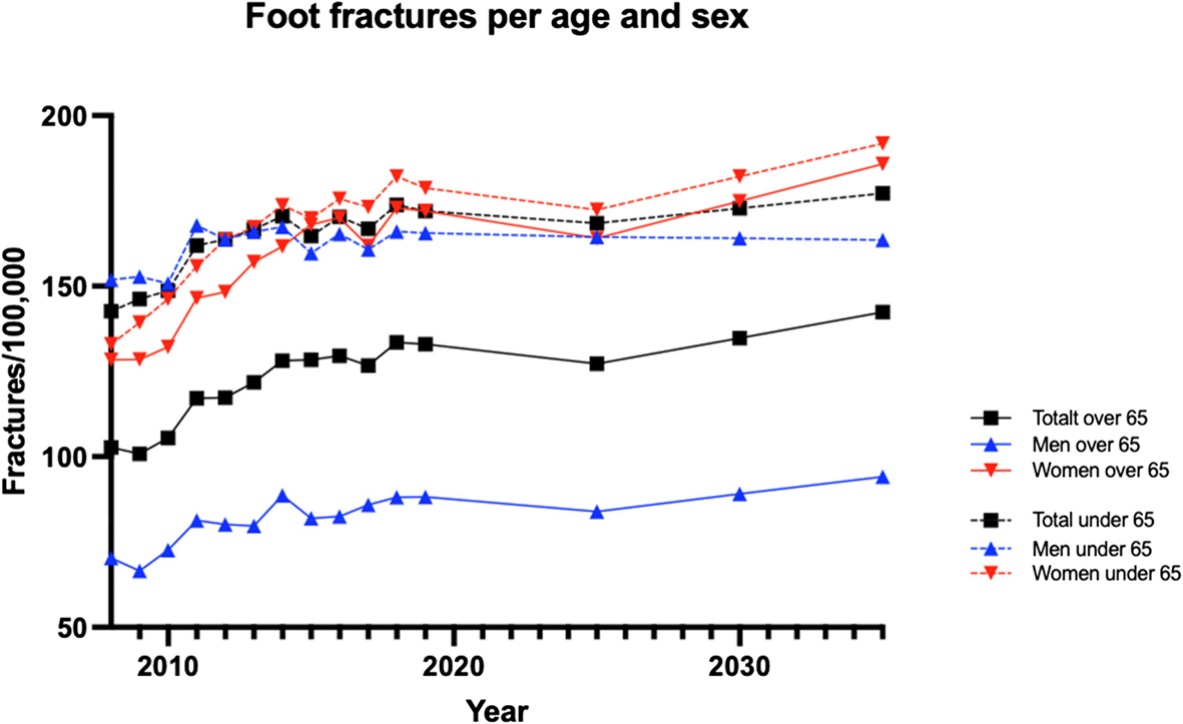
Projection of the incidence of foot fractures in men and women per age group excluding the pandemic years of 2020–2021

To account for pandemic influence, we also analyzed the incidence of foot fractures starting from 2008 but excluded the years 2020–2021 in the projection analysis. The anticipated trends in foot fracture rates from 2019 to 2035 include a potential 7% increase in foot fractures for men over 65, a slight 1% decrease for men under 65, a significant 8% increase for women over 65, a 7% increase for women under 65 (Fig. 6).

## Discussion

This study aimed to describe trends, seasonal variations, and future projections in foot fracture incidence in Sweden using extensive data from the SNBHW. Noteworthy findings include a significant drop in foot fracture incidence in 2020, age and sex disparities influencing fracture rates, and a compelling projection analysis foreseeing changes in rates from 2019 to 2035.

The average annual foot fracture incidence between 2008 and 2022 was 11,942 cases. A significant drop in incidence occurred in 2020, likely influenced by the COVID-19 pandemic, with a subsequent rebound in 2021–2022. This pattern varied among age and sex groups, with a noteworthy reduction in fracture incidence during 2020 for women over 65 and men under 65. We noted fairly low year-to-year changes during the study period, this is in line with similar studies [21–23].

Examining age-specific incidence rates revealed distinct patterns. Women demonstrated a peak incidence of 218/100,000 in the 45–64 age group, while men experienced the highest incidence (140/100,000) in the 18–29 age group. This is in line with studies focusing on the epidemiology of foot fractures in similar countries [24–26]. These findings highlight age and sex as crucial factors influencing foot fracture rates.

Seasonal analysis showed that foot fracture incidence increases during summer months. This seasonal variance was consistent across age groups, with 40% of fractures occurring in summer, emphasizing the importance of seasonal considerations in healthcare planning. Other factors which could influence incidence include demographic shifts caused by an aging population, improved traffic safety, increased lifelong physical activity which could contribute to alterations in incidence rates, trauma mechanisms, fracture types, and the distribution of fractures among different genders and age groups. Additionally, various studies addressing the incidence of fractures in the lower extremities have highlighted evolving patterns over time [27–29]. Our projection analysis anticipates changes in foot fracture rates from 2019 to 2035. Notable projections include a potential decrease in rates for men over 65 and men under 65, a significant increase for women over 65, and a moderate increase for women under 65.

The influence of the COVID-19 pandemic on orthopaedic pathologies is currently being evaluated. The number of elective procedures for both hip and knee decreased during the pandemic which has raised question regarding the implementation of targeted interventions such as new rehab protocols and clinical solutions [30, 31]. Furthermore, elective orthopaedic surgeries during the pandemic has been linked to both increased risk of readmission and increased mortality [32]. COVID-19 infection is also associated with increased mortality and complication in trauma patients [33, 34]. Indirect effects such as increased waiting times due to testing and disruptions to hip fracture services might influence the risk of complications for orthopaedic patients during pandemic conditions [35–37]. The impact of the COVID-19 was not uniform on a global scale as studies from different countries report varying decreases in orthopaedic services [38–41].

There is currently a lack of larger nationwide studies which examines the influence of the COVID-19 pandemic on traumatic lower limb orthopaedic conditions such as foot fractures. Smaller studies form the United Kingdom, USA, Turkey and Italy found decreasing foot fractures incidence, in similarity with our results, during the pandemic years [42–46]. It is likely that foot fracture incidence will mimic the pattern of other fractures such as upper limb fractures where reports show variances between countries associated to pandemic response and infection levels [47–49].

This study highlights significant trends and disparities in foot fracture incidence in Sweden, which we hope might serve as a base for future research and healthcare planning. The observed seasonal and demographic variations indicate a need for targeted preventive measures and resource allocation strategies, especially for high-risk groups like the elderly and women. Projections indicating potential decreases in foot fracture rates among several demographic groups by 2035 where improvements in traffic safety, physical activity, and preventive healthcare could be effective. However, the projected increase in fractures among younger women underscores the importance of continued surveillance for this group. Future studies should focus on understanding the underlying causes of these trends, evaluating the impact of preventive strategies, and developing tailored interventions to mitigate fracture risk across different population groups. Additionally, comprehending the long-term effects of the COVID-19 pandemic on orthopedic injuries will be crucial for adapting healthcare services and policies to ensure resilience against similar disruptions in the future.

This study comes with certain limitations. Firstly, reliance on publicly aggregated data introduces constraints, preventing the differentiation of subgroups within the ICD code of S92, such as specific distinctions for talus fractures or individual patient identification. Publicly aggregated data may also be prone to under-/overreporting or coding inaccuracies, although ongoing efforts by the SNBHW through regular audits and validations aim to enhance data quality. Secondly, the lack of detailed clinical information, as individual patients remain unidentified, hinders exploration into factors like the mechanism of injury, comorbidities, osteoporosis, physical activity levels, and socioeconomic factors. This absence limits our ability to investigate potential risk factors or comprehend the context of foot fractures within the population. Despite these constraints, the study offers valuable insights into the temporal trends, sex differences, and seasonal variations in foot fractures within the Swedish population, utilizing publicly aggregated data.

## Conclusion

This study, utilizing comprehensive data and advanced analytical methods, sheds light on the multifaceted landscape of foot fractures in Sweden. The identified trends and projections offer a foundation for targeted preventive strategies, efficient resource allocation, and informed healthcare planning. Elderly people will experience an increase in foot fracture incidence if current trends continue. The findings contribute to the broader understanding of orthopedic injuries, emphasizing the need for adaptive healthcare strategies to address evolving patterns and challenges.

## Data Availability

The data sets can be obtained from the NPR directly (https://www.socialstyrelsen.se/en/statistics-and-data/statistics/statistical-databases/).

## Abbreviations

ICD-10: International Classification of Diseases, Tenth Revision
NPR: National Patient Register
SNBHW: Swedish National Board of Health and Welfare
SPSS: Statistical Package for the Social Sciences
